# The crystal structure of 2-[5-(di­methyl­amino)­naphthalene-1-sulfonamido]­phenyl 5-(di­methyl­amino)­naphthalene-1-sulfonate

**DOI:** 10.1107/S2056989015016199

**Published:** 2015-09-12

**Authors:** Kittipong Chainok, Tanwawan Duangthongyou, Thawatchai Tuntulani, Apinya Chuenka, Boontana Wannalerse

**Affiliations:** a Department of Physics, Faculty of Science and Technology, Thammasat University, Khlong Luang, Pathum Thani, 12120, Thailand; b Department of Chemistry, Faculty of Science, Kasetsart University, Bangkok 10903, Thailand; c Supramolecular Chemistry Research Unit, Department of Chemistry, Faculty of Science, Chulalongkorn University, Bangkok 10330, Thailand; d Department of Chemistry and Center of Excellence for Innovation in Chemistry, Faculty of Science, Kasetsart University, Bangkok 10903, Thailand

**Keywords:** crystal structure, dansyl derivatives, disorder, hydrogen bonding, π-stacking

## Abstract

The complete mol­ecule of the title compound, C_30_H_29_N_3_O_5_S_2_, is generated by a crystallographic twofold axis: the O atom and NH group attached to the central benzene ring are statistically disordered. The dihedral angle between the naphthalene ring system and the central benzene ring is 52.99 (6)°, while the pendant naphthalene ring systems subtend a dihedral angle of 68.17 (4)°. An intra­molecular C—H⋯O hydrogen bond closes an *S*(6) ring. In the crystal, the mol­ecules are linked by weak C—H⋯O hydrogen bonds.

## Related literature   

For the use of dansyl tags to monitor biological activity in enzyme systems, see: Brown *et al.* (1970 ▸); Liu *et al.* (2010 ▸). Dansyl-conjugated liposome has been used to modulate the fluorescence resonance energy transfer (FRET) mechanism, see: Li *et al.* (2006 ▸). Dansyl fluoro­genic sensors have been used for the recognition and detection of targets such as cationic and anionic species, see: Cao *et al.* (2014 ▸); Jisha *et al.* (2009 ▸); Bhalla *et al.* (2007 ▸). For crystal structures of dansyl derivatives, see: Bhatt *et al.* (2011 ▸); Zhang *et al.* (2009 ▸) and of metal–calix[4]arene complexes bearing two dansyl carboxamide units, see: Buie *et al.* (2008 ▸).
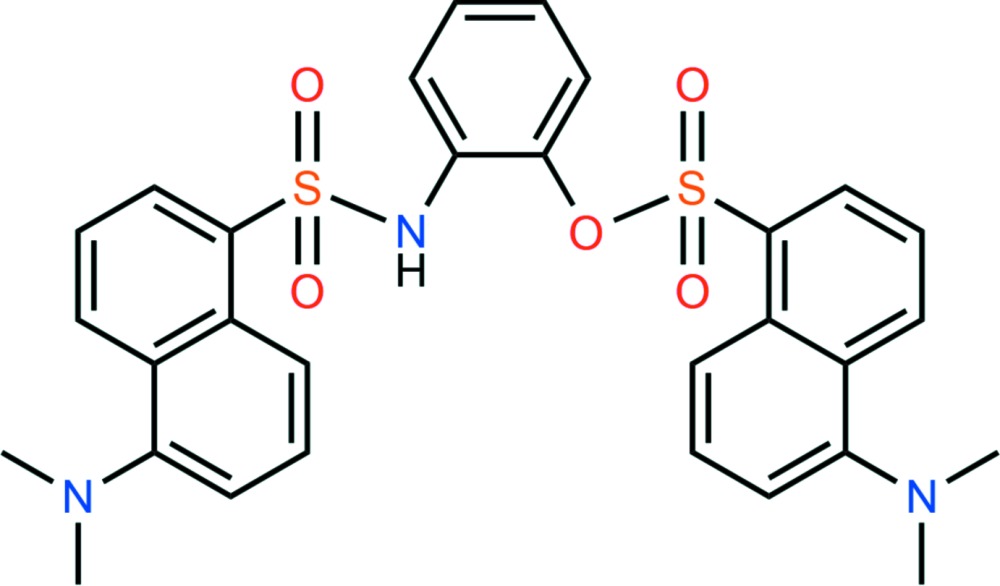


## Experimental   

### Crystal data  

C_30_H_29_N_3_O_5_S_2_
*M* *_r_* = 575.68
Monoclinic, 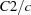

*a* = 12.7594 (13) Å
*b* = 13.3481 (14) Å
*c* = 16.4331 (17) Å
β = 98.349 (4)°
*V* = 2769.1 (5) Å^3^
*Z* = 4
Mo *K*α radiation
μ = 0.24 mm^−1^
*T* = 296 K
0.26 × 0.22 × 0.22 mm

### Data collection  

Bruker D8 QUEST CMOS diffractometer
Absorption correction: multi-scan (*SADABS*; Bruker, 2014 ▸) *T*_min_ = 0.698, *T*_max_ = 0.746
17644 measured reflections
3444 independent reflections
2246 reflections with *I* > 2σ(*I*)
*R*_int_ = 0.041

### Refinement  

*R*[*F* ^2^ > 2σ(*F* ^2^)] = 0.047
*wR*(*F* ^2^) = 0.113
*S* = 1.03
3444 reflections
186 parameters
H-atom parameters constrained
Δρ_max_ = 0.26 e Å^−3^
Δρ_min_ = −0.23 e Å^−3^

### 

Data collection: *APEX2* (Bruker, 2014 ▸); cell refinement: *SAINT* (Bruker, 2014 ▸); data reduction: *SAINT*; program(s) used to solve structure: *SHELXT* (Sheldrick, 2015*a* ▸); program(s) used to refine structure: *SHELXL2014* (Sheldrick, 2015*b* ▸); molecular graphics: *OLEX2* (Dolomanov *et al.*, 2009 ▸); software used to prepare material for publication: *OLEX2*.

## Supplementary Material

Crystal structure: contains datablock(s) I. DOI: 10.1107/S2056989015016199/gw2153sup1.cif

Structure factors: contains datablock(s) bw6. DOI: 10.1107/S2056989015016199/gw2153Isup2.hkl

Supporting information file. DOI: 10.1107/S2056989015016199/gw2153Isup3.cdx

Supporting information file. DOI: 10.1107/S2056989015016199/gw2153Isup4.cml

**Fig. 1.**
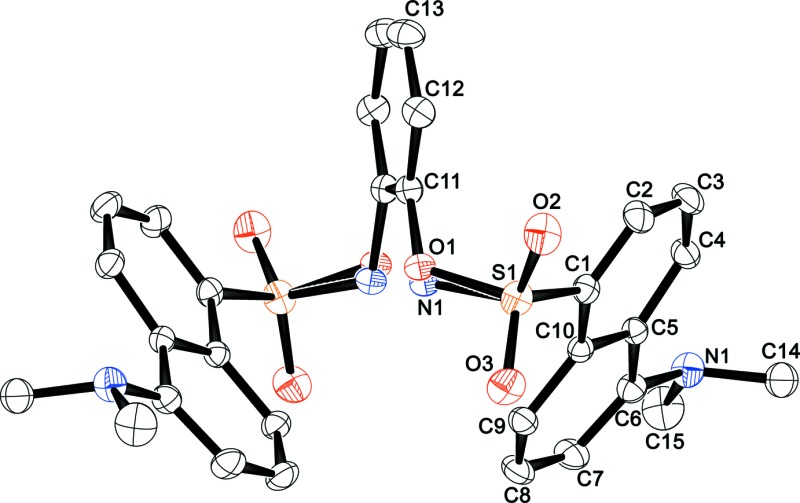
The molecular structure of the title compound with 30% probability ellipsoids and atom numbering. Hydrogen atoms are omitted for clarity.

. DOI: 10.1107/S2056989015016199/gw2153fig1.tif
The mol­ecular structure of the title compound with 30% probability ellipsoids and atom numbering. Hydrogen atoms are omitted for clarity.

**Fig. 2.**
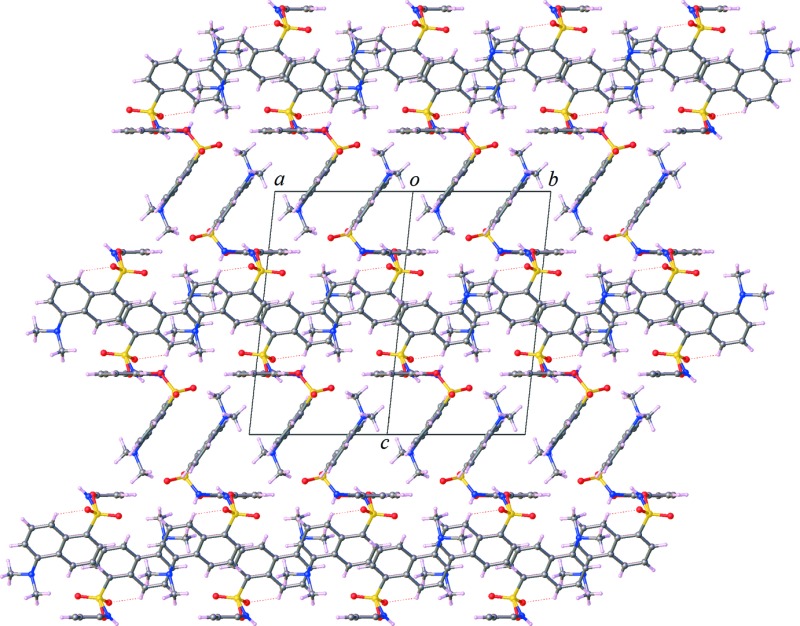
The crystal packing of the title compound, viewed along the [110] direction.

. DOI: 10.1107/S2056989015016199/gw2153fig2.tif
The crystal packing of the title compound, viewed along the [110] direction.

CCDC reference: 1421273

Additional supporting information:  crystallographic information; 3D view; checkCIF report

## Figures and Tables

**Table 1:** Hydrogen-bond geometry (, )

*D*H*A*	*D*H	H*A*	*D* *A*	*D*H*A*
C7H7O3	0.93	2.37	3.030(2)	128
C13H13O3^i^	0.93	2.73	3.386(2)	129

Symmetry code: (i) 
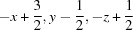
.

## supplementary crystallographic information

### S1. Introduction

Dansyl derivatives can be widely used as fluorescence probes in biological and environmental systems. Dansyl tags have been increasingly used to monitor biological activities in the enzyme system for providing the accurate information (Brown *et al.*, 1970; Liu *et al.*, 2010). An example is dansyl-conjugated liposome for modulating fluorescence resonance energy transfer (FRET) mechanism (Li *et al.* 2006). Furthermore, dansyl fluoro­genic sensors were prepared for recognition and detection of many targets such as cationic and anionic species (Cao *et al.*, 2014; Jisha *et al.*, 2009; Bhalla *et al.*, 2007). Crystal structures of dansyl derivatives (Bhatt *et al.*, 2011; Zhang *et al.*, 2009) and metal complexes of calix[4]arene bearing two dansyl carboxamide units have been reported (Buie *et al.*, 2008).

### S2. Synthesis and crystallization

The title compound was synthesized by condensation of 2-amino­phenol (0.55 g, 5.04 mmol) and dansyl chloride (2.72 g, 10.08 mmol) using potassium carbonate (17.27 g, 12.50 mmol) as a base in aceto­nitrile (30 ml). The solution was heated and stirred under N_2_ atmosphere for 24 h. The solvent was then removed by a rotary evaporator. Water (10 ml) was added to the residue and the organic phase was extracted with di­chloro­methane (3 x 20 ml). The organic layer was dried with Na_2_SO_4_. The product was purified by column chromatography using di­chloro­methane as an eluent. The solvent was evaporated to afford a yellow crystalline solid in 55% yield. Single crystals suitable for X-ray measurements were obtained by recrystallization using the mixture solution of di­chloro­methane and hexane (1:1, *v*/*v*) at room temperature.

### S3. Refinement

Atom O1 and the N1H1 group attached to the central benzene ring are statistically disordered and were refined with the occupancies of the N, H and O atoms fixed at 0.5. All H atoms were positioned geometrically and refined using a riding model, with C—H = 0.93 Å for aryl and 0.96 Å for methyl H atoms, *U*_iso_ (H) = 1.2*U*_eq_ (C) for aryl and 1.5*U*_eq_ (C) for methyl H atoms. The N-bound H-atom was refined with N—H = 0.86 Å, and with *U*_iso_(H) = 1.2*U*_eq_(N).

### Crystal data

**Table:**

C_30_H_29_N_3_O_5_S_2_	*F*(000) = 1208
*M**_r_* = 575.68	*D*_x_ = 1.381 Mg m^−^^3^
Monoclinic, *C*2/*c*	Mo *K*α radiation, λ = 0.71073 Å
*a* = 12.7594 (13) Å	Cell parameters from 4957 reflections
*b* = 13.3481 (14) Å	θ = 3.1–25.7°
*c* = 16.4331 (17) Å	µ = 0.24 mm^−^^1^
β = 98.349 (4)°	*T* = 296 K
*V* = 2769.1 (5) Å^3^	Block, light green
*Z* = 4	0.26 × 0.22 × 0.22 mm

### Data collection

**Table:**

Bruker D8 QUEST CMOS diffractometer	3444 independent reflections
Radiation source: microfocus sealed x-ray tube, Incoatec Iµus	2246 reflections with *I* > 2σ(*I*)
GraphiteDouble Bounce Multilayer Mirror monochromator	*R*_int_ = 0.041
Detector resolution: 10.5 pixels mm^-1^	θ_max_ = 28.3°, θ_min_ = 3.1°
ω and φ scans	*h* = −15→16
Absorption correction: multi-scan (*SADABS*; Bruker, 2014)	*k* = −17→17
*T*_min_ = 0.698, *T*_max_ = 0.746	*l* = −21→21
17644 measured reflections	

### Refinement

**Table:**

Refinement on *F*^2^	Primary atom site location: structure-invariant direct methods
Least-squares matrix: full	Hydrogen site location: inferred from neighbouring sites
*R*[*F*^2^ > 2σ(*F*^2^)] = 0.047	H-atom parameters constrained
*wR*(*F*^2^) = 0.113	*w* = 1/[σ^2^(*F*_o_^2^) + (0.0488*P*)^2^ + 1.1669*P*] where *P* = (*F*_o_^2^ + 2*F*_c_^2^)/3
*S* = 1.03	(Δ/σ)_max_ < 0.001
3444 reflections	Δρ_max_ = 0.26 e Å^−^^3^
186 parameters	Δρ_min_ = −0.23 e Å^−^^3^
0 restraints	

### Special details

**Table:**

Geometry. All e.s.d.'s (except the e.s.d. in the dihedral angle between two l.s. planes) are estimated using the full covariance matrix. The cell e.s.d.'s are taken into account individually in the estimation of e.s.d.'s in distances, angles and torsion angles; correlations between e.s.d.'s in cell parameters are only used when they are defined by crystal symmetry. An approximate (isotropic) treatment of cell e.s.d.'s is used for estimating e.s.d.'s involving l.s. planes.

### Fractional atomic coordinates and isotropic or equivalent isotropic displacement parameters (Å^2^)

**Table:**

	*x*	*y*	*z*	*U*_iso_*/*U*_eq_	Occ. (<1)
S1	0.69732 (3)	0.73580 (4)	0.32645 (3)	0.05431 (17)	
N1	0.603 (2)	0.709 (2)	0.2569 (14)	0.0418 (18)	0.5
H1	0.5801	0.7537	0.2215	0.050*	0.5
O2	0.77825 (10)	0.66139 (12)	0.33521 (10)	0.0736 (5)	
O3	0.72349 (11)	0.83601 (11)	0.30878 (10)	0.0711 (4)	
N2	0.35617 (13)	0.82675 (13)	0.57539 (10)	0.0609 (5)	
C11	0.55450 (13)	0.61136 (13)	0.25005 (10)	0.0433 (4)	
C5	0.49120 (13)	0.77874 (12)	0.49182 (10)	0.0427 (4)	
C6	0.54736 (13)	0.79731 (12)	0.42414 (10)	0.0413 (4)	
C1	0.62854 (13)	0.72779 (13)	0.41220 (11)	0.0450 (4)	
C10	0.40779 (14)	0.84536 (14)	0.50673 (12)	0.0495 (4)	
C12	0.60785 (15)	0.52183 (15)	0.24889 (12)	0.0543 (5)	
H12	0.6806	0.5215	0.2481	0.065*	
C7	0.51636 (15)	0.87928 (13)	0.37174 (11)	0.0500 (4)	
H7	0.5533	0.8940	0.3285	0.060*	
C4	0.51413 (16)	0.69084 (14)	0.53896 (12)	0.0531 (5)	
H4	0.4748	0.6766	0.5810	0.064*	
C2	0.65034 (16)	0.64565 (15)	0.46148 (12)	0.0582 (5)	
H2	0.7046	0.6022	0.4527	0.070*	
C9	0.37880 (17)	0.92039 (15)	0.45129 (13)	0.0612 (5)	
H9	0.3220	0.9615	0.4583	0.073*	
C8	0.43259 (17)	0.93625 (15)	0.38477 (13)	0.0611 (5)	
H8	0.4104	0.9876	0.3480	0.073*	
C13	0.55357 (17)	0.43329 (16)	0.24897 (14)	0.0665 (6)	
H13	0.5895	0.3728	0.2476	0.080*	
C3	0.59130 (17)	0.62698 (15)	0.52481 (13)	0.0635 (6)	
H3	0.6052	0.5702	0.5574	0.076*	
C14	0.41854 (19)	0.83896 (17)	0.65611 (13)	0.0707 (6)	
H14A	0.4905	0.8197	0.6537	0.106*	
H14B	0.3898	0.7975	0.6951	0.106*	
H14C	0.4165	0.9078	0.6727	0.106*	
C15	0.25143 (19)	0.8715 (2)	0.57308 (17)	0.0878 (8)	
H15A	0.2587	0.9419	0.5846	0.132*	
H15B	0.2153	0.8401	0.6136	0.132*	
H15C	0.2114	0.8620	0.5195	0.132*	
O1	0.6121 (16)	0.7010 (16)	0.2444 (11)	0.0418 (18)	0.5

### Atomic displacement parameters (Å^2^)

**Table:**

	*U*^11^	*U*^22^	*U*^33^	*U*^12^	*U*^13^	*U*^23^
S1	0.0322 (2)	0.0701 (4)	0.0622 (3)	−0.0032 (2)	0.0121 (2)	−0.0040 (3)
N1	0.035 (3)	0.051 (3)	0.043 (5)	0.0008 (19)	0.017 (2)	−0.002 (3)
O2	0.0366 (7)	0.1019 (12)	0.0833 (10)	0.0177 (7)	0.0120 (7)	−0.0060 (9)
O3	0.0528 (8)	0.0783 (10)	0.0848 (10)	−0.0260 (7)	0.0188 (7)	−0.0017 (8)
N2	0.0564 (10)	0.0708 (12)	0.0595 (10)	0.0057 (8)	0.0213 (8)	−0.0053 (8)
C11	0.0430 (9)	0.0499 (10)	0.0401 (9)	−0.0004 (8)	0.0161 (8)	0.0007 (8)
C5	0.0422 (9)	0.0435 (10)	0.0411 (9)	0.0018 (7)	0.0023 (7)	−0.0002 (7)
C6	0.0387 (9)	0.0416 (9)	0.0426 (9)	−0.0021 (7)	0.0023 (7)	−0.0026 (7)
C1	0.0367 (9)	0.0517 (10)	0.0454 (10)	0.0024 (8)	0.0017 (7)	−0.0020 (8)
C10	0.0474 (10)	0.0508 (11)	0.0509 (11)	0.0029 (8)	0.0088 (8)	−0.0037 (8)
C12	0.0515 (11)	0.0591 (13)	0.0566 (11)	0.0084 (9)	0.0225 (9)	0.0023 (9)
C7	0.0572 (11)	0.0458 (10)	0.0478 (10)	0.0015 (9)	0.0101 (9)	0.0054 (8)
C4	0.0616 (12)	0.0523 (11)	0.0459 (10)	0.0048 (9)	0.0098 (9)	0.0076 (9)
C2	0.0543 (11)	0.0605 (12)	0.0585 (12)	0.0223 (9)	0.0033 (9)	0.0026 (10)
C9	0.0606 (12)	0.0543 (12)	0.0702 (13)	0.0212 (10)	0.0144 (10)	0.0039 (10)
C8	0.0715 (14)	0.0484 (11)	0.0626 (12)	0.0162 (10)	0.0074 (10)	0.0128 (9)
C13	0.0791 (14)	0.0509 (12)	0.0752 (14)	0.0108 (10)	0.0306 (13)	0.0021 (11)
C3	0.0775 (14)	0.0554 (12)	0.0570 (12)	0.0212 (11)	0.0078 (11)	0.0144 (10)
C14	0.0904 (17)	0.0706 (15)	0.0549 (13)	0.0006 (12)	0.0233 (12)	−0.0004 (11)
C15	0.0640 (14)	0.111 (2)	0.0949 (19)	0.0165 (14)	0.0343 (14)	−0.0055 (15)
O1	0.035 (3)	0.051 (3)	0.043 (5)	0.0008 (19)	0.017 (2)	−0.002 (3)

### Geometric parameters (Å, º)

**Table:**

S1—N1	1.58 (3)	C12—H12	0.9300
S1—O2	1.4248 (14)	C12—C13	1.370 (3)
S1—O3	1.4189 (15)	C7—H7	0.9300
S1—C1	1.7681 (18)	C7—C8	1.354 (3)
S1—O1	1.67 (2)	C4—H4	0.9300
N1—H1	0.8600	C4—C3	1.348 (3)
N1—C11	1.44 (3)	C2—H2	0.9300
N2—C10	1.409 (2)	C2—C3	1.393 (3)
N2—C14	1.454 (3)	C9—H9	0.9300
N2—C15	1.459 (3)	C9—C8	1.389 (3)
C11—C11^i^	1.391 (3)	C8—H8	0.9300
C11—C12	1.377 (2)	C13—C13^i^	1.372 (4)
C11—O1	1.41 (2)	C13—H13	0.9300
C5—C6	1.429 (2)	C3—H3	0.9300
C5—C10	1.435 (2)	C14—H14A	0.9600
C5—C4	1.413 (2)	C14—H14B	0.9600
C6—C1	1.425 (2)	C14—H14C	0.9600
C6—C7	1.413 (2)	C15—H15A	0.9600
C1—C2	1.367 (3)	C15—H15B	0.9600
C10—C9	1.368 (3)	C15—H15C	0.9600
			
N1—S1—C1	98.6 (8)	C6—C7—H7	120.1
O2—S1—N1	112.1 (9)	C8—C7—C6	119.74 (17)
O2—S1—C1	108.28 (9)	C8—C7—H7	120.1
O2—S1—O1	105.4 (6)	C5—C4—H4	119.0
O3—S1—N1	104.3 (10)	C3—C4—C5	121.93 (18)
O3—S1—O2	119.29 (9)	C3—C4—H4	119.0
O3—S1—C1	112.27 (9)	C1—C2—H2	120.0
O3—S1—O1	104.0 (7)	C1—C2—C3	120.02 (18)
O1—S1—C1	106.6 (6)	C3—C2—H2	120.0
S1—N1—H1	118.6	C10—C9—H9	119.4
C11—N1—S1	122.8 (18)	C10—C9—C8	121.27 (18)
C11—N1—H1	118.6	C8—C9—H9	119.4
C10—N2—C14	116.97 (16)	C7—C8—C9	122.05 (18)
C10—N2—C15	116.09 (18)	C7—C8—H8	119.0
C14—N2—C15	110.84 (18)	C9—C8—H8	119.0
C11^i^—C11—N1	115.0 (10)	C12—C13—C13^i^	120.37 (12)
C11^i^—C11—O1	121.9 (8)	C12—C13—H13	119.8
C12—C11—N1	125.1 (10)	C13^i^—C13—H13	119.8
C12—C11—C11^i^	119.76 (11)	C4—C3—C2	120.24 (18)
C12—C11—O1	118.1 (8)	C4—C3—H3	119.9
C6—C5—C10	119.56 (15)	C2—C3—H3	119.9
C4—C5—C6	118.90 (16)	N2—C14—H14A	109.5
C4—C5—C10	121.37 (17)	N2—C14—H14B	109.5
C1—C6—C5	116.78 (15)	N2—C14—H14C	109.5
C7—C6—C5	118.75 (16)	H14A—C14—H14B	109.5
C7—C6—C1	124.40 (16)	H14A—C14—H14C	109.5
C6—C1—S1	121.67 (13)	H14B—C14—H14C	109.5
C2—C1—S1	116.02 (14)	N2—C15—H15A	109.5
C2—C1—C6	122.00 (17)	N2—C15—H15B	109.5
N2—C10—C5	118.17 (16)	N2—C15—H15C	109.5
C9—C10—N2	123.36 (17)	H15A—C15—H15B	109.5
C9—C10—C5	118.38 (17)	H15A—C15—H15C	109.5
C11—C12—H12	120.1	H15B—C15—H15C	109.5
C13—C12—C11	119.85 (18)	C11—O1—S1	117.7 (13)
C13—C12—H12	120.1		
			
S1—N1—C11—C11^i^	124.2 (13)	C6—C5—C4—C3	3.7 (3)
S1—N1—C11—C12	−51.6 (18)	C6—C1—C2—C3	1.1 (3)
S1—C1—C2—C3	−172.60 (16)	C6—C7—C8—C9	3.7 (3)
N1—S1—C1—C6	−67.6 (10)	C1—S1—N1—C11	−66.4 (15)
N1—S1—C1—C2	106.1 (10)	C1—S1—O1—C11	−48.7 (12)
N1—C11—C12—C13	174.6 (11)	C1—C6—C7—C8	174.71 (18)
O2—S1—N1—C11	47.5 (16)	C1—C2—C3—C4	−1.5 (3)
O2—S1—C1—C6	175.57 (14)	C10—C5—C6—C1	−179.37 (15)
O2—S1—C1—C2	−10.74 (18)	C10—C5—C6—C7	−2.3 (3)
O2—S1—O1—C11	66.2 (11)	C10—C5—C4—C3	179.07 (19)
O3—S1—N1—C11	177.9 (12)	C10—C9—C8—C7	−0.5 (3)
O3—S1—C1—C6	41.79 (17)	C12—C11—O1—S1	−72.7 (11)
O3—S1—C1—C2	−144.52 (15)	C7—C6—C1—S1	−2.0 (2)
O3—S1—O1—C11	−167.5 (9)	C7—C6—C1—C2	−175.27 (19)
N2—C10—C9—C8	179.6 (2)	C4—C5—C6—C1	−4.0 (2)
C11^i^—C11—C12—C13	−1.0 (3)	C4—C5—C6—C7	173.16 (17)
C11^i^—C11—O1—S1	112.6 (11)	C4—C5—C10—N2	6.6 (3)
C11—C12—C13—C13^i^	−0.6 (4)	C4—C5—C10—C9	−170.03 (19)
C5—C6—C1—S1	174.98 (12)	C14—N2—C10—C5	66.8 (2)
C5—C6—C1—C2	1.7 (3)	C14—N2—C10—C9	−116.8 (2)
C5—C6—C7—C8	−2.2 (3)	C15—N2—C10—C5	−159.33 (19)
C5—C10—C9—C8	−4.0 (3)	C15—N2—C10—C9	17.1 (3)
C5—C4—C3—C2	−1.0 (3)	O1—S1—C1—C6	−71.5 (8)
C6—C5—C10—N2	−178.08 (16)	O1—S1—C1—C2	102.2 (8)
C6—C5—C10—C9	5.3 (3)	O1—C11—C12—C13	−175.8 (9)

Symmetry code: (i) −*x*+1, *y*, −*z*+1/2.

### Hydrogen-bond geometry (Å, º)

**Table:**

*D*—H···*A*	*D*—H	H···*A*	*D*···*A*	*D*—H···*A*
C7—H7···O3	0.93	2.37	3.030 (2)	128
C13—H13···O3^ii^	0.93	2.73	3.386 (2)	129

Symmetry code: (ii) −*x*+3/2, *y*−1/2, −*z*+1/2.
