# Supplementary figures and images for: The crystal structure of 2-[5-(di­methyl­amino)­naphthalene-1-sulfonamido]­phenyl 5-(di­methyl­amino)­naphthalene-1-sulfonate
Source: Acta Crystallogr E Crystallogr Commun. 2015 Sep 12;71(Pt 10):o721–2. doi: 10.1107/S2056989015016199 (PMC4647440; doi:10.1107/S2056989015016199)

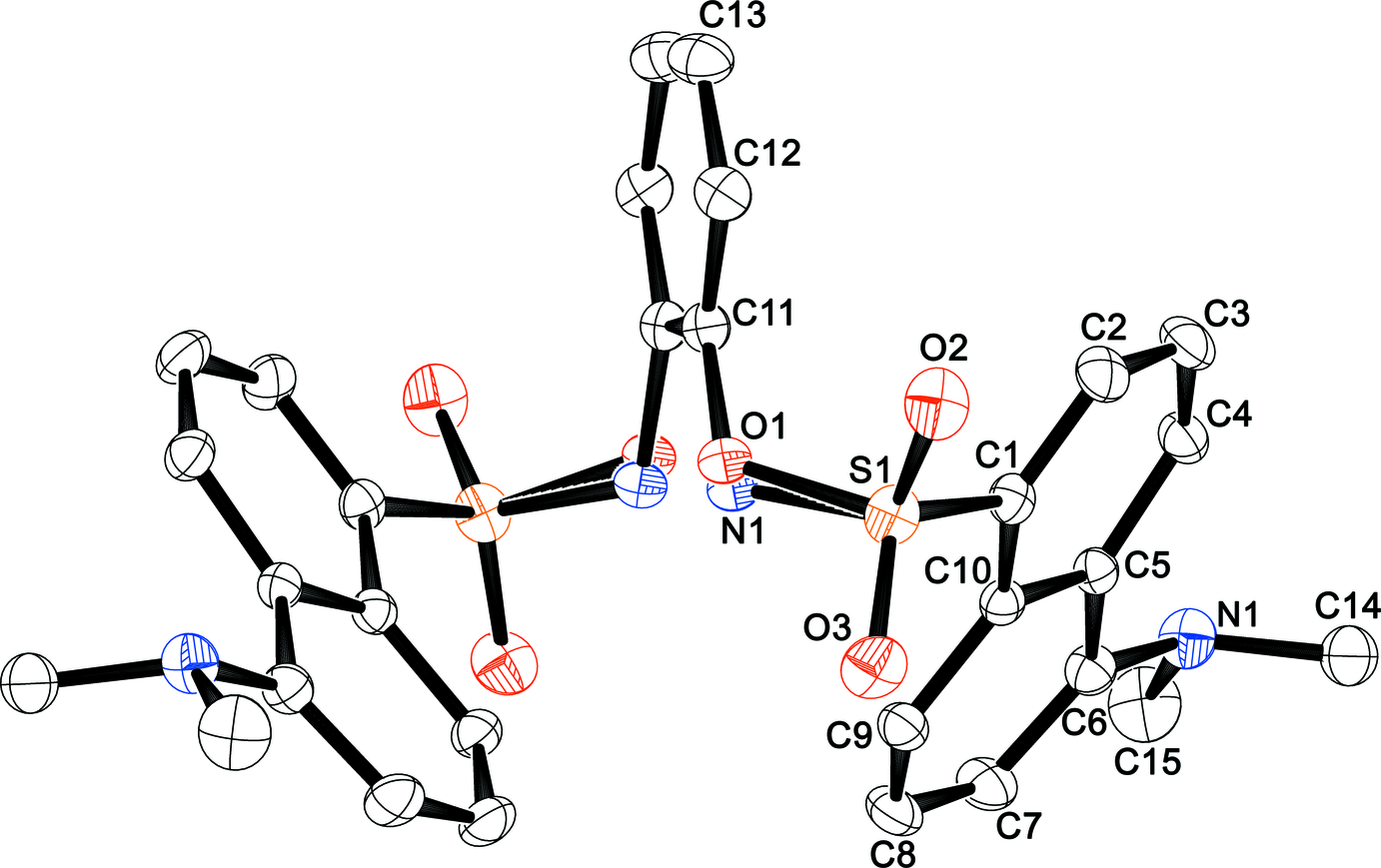

Supplement: Supplementary file 5 [file e-71-0o721-fig1.tif]

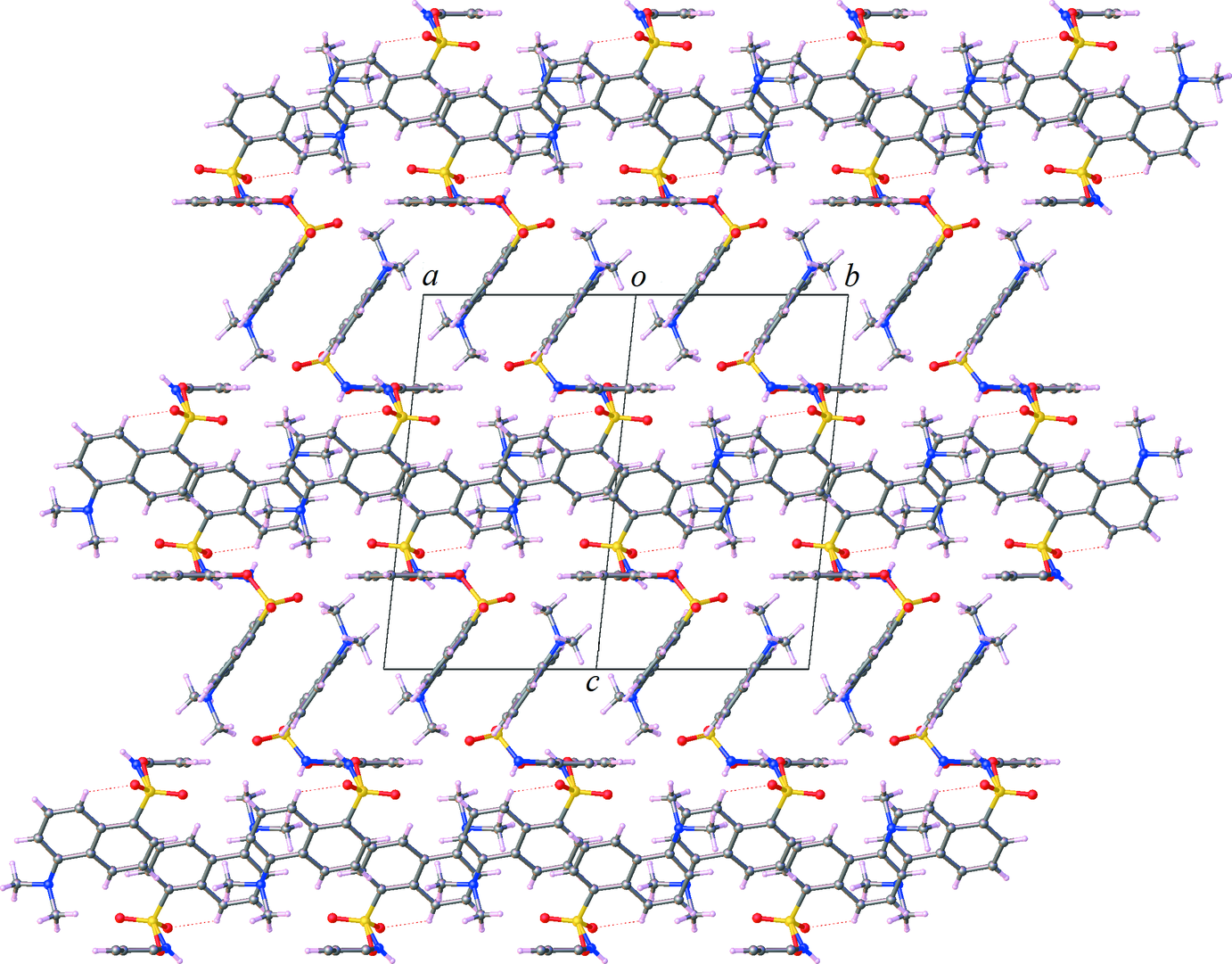

Supplement: Supplementary file 6 [file e-71-0o721-fig2.tif]
